# Expression, intracellular targeting and purification of HIV Nef variants in tobacco cells

**DOI:** 10.1186/1472-6750-7-12

**Published:** 2007-02-26

**Authors:** Carla Marusic, James Nuttall, Giampaolo Buriani, Chiara Lico, Raffaele Lombardi, Selene Baschieri, Eugenio Benvenuto, Lorenzo Frigerio

**Affiliations:** 1 ENEA-BIOTEC Sezione Genetica e Genomica Vegetale, C.R. Casaccia, 00060 Rome, Italy; 2 Department of Biological Sciences, University of Warwick, Coventry CV4 7AL, UK

## Abstract

**Background:**

Plants may represent excellent alternatives to classical heterologous protein expression systems, especially for the production of biopharmaceuticals and vaccine components. Modern vaccines are becoming increasingly complex, with the incorporation of multiple antigens. Approaches towards developing an HIV vaccine appear to confirm this, with a combination of candidate antigens. Among these, HIV-Nef is considered a promising target for vaccine development because immune responses directed against this viral protein could help to control the initial steps of viral infection and to reduce viral loads and spreading. Two isoforms of Nef protein can be found in cells: a full-length N-terminal myristoylated form (p27, 27 kDa) and a truncated form (p25, 25 kDa). Here we report the expression and purification of HIV Nef from transgenic tobacco.

**Results:**

We designed constructs to direct the expression of p25 and p27 Nef to either the cytosol or the secretory pathway. We tested these constructs by transient expression in tobacco protoplasts. Cytosolic Nef polypeptides are correctly synthesised and are stable. The same is not true for Nef polypeptides targeted to the secretory pathway by virtue of a signal peptide. We therefore generated transgenic plants expressing cytosolic, full length or truncated Nef. Expression levels were variable, but in some lines they averaged 0.7% of total soluble proteins. Hexahistidine-tagged Nef was easily purified from transgenic tissue in a one-step procedure.

**Conclusion:**

We have shown that transient expression can help to rapidly determine the best cellular compartment for accumulation of a recombinant protein. We have successfully expressed HIV Nef polypeptides in the cytosol of transgenic tobacco plants. The proteins can easily be purified from transgenic tissue.

## Background

Plants have emerged as a safe and economical alternative to mainstream protein expression systems based on the large-scale culture of microbes or animal cells or on transgenic animals to produce biopharmaceuticals. Diverse, complex macromolecules such as antibodies [1,2] and vaccine components [3] have been successfully expressed in plant cells. The possibility to produce biopharmaceuticals using plants offers solutions to some of the problems associated to traditional heterologous expression systems. For example, the bacterial production of biologically active, complex multimeric proteins such as antibodies is limited by the absence of the enzymatic machinery involved in post-translational modification of newly-synthesised proteins [1,2]. Among eukaryotic expression systems, yeast is not always appropriate as hyperglycosylation of the final product is often encountered, even if several laboratories are in the process of modulating glycosylation pathways to obtain humanized yeast-derived glycoproteins [4,5]. Insect and mammalian cell cultures represent complex expression platforms requiring expensive procedures and may be easily contaminated with toxins, viruses or prions, raising concerns on the safety of the final product. The plant secretory pathway, on the other hand, has been shown to be particularly suitable for the production and accumulation of high amounts of heterologous proteins [6,7].

Modern vaccines are becoming increasingly complex, with several constituted by a combination of multiple antigens. Most of the current strategies for vaccination against HIV/AIDS involve targeting a combination of HIV and host antigens [8]. Plant-based expression of a number of these candidates has already been achieved, including HIV-1 gp120 envelope glycoprotein [8], p24 core protein [9] and the regulatory Tat protein [10].

Both regulatory and accessory HIV proteins are currently regarded as promising targets for vaccine development as they could provide further protective efficacy in combination with viral structural proteins. For this purpose, HIV-1 accessory Nef protein is considered a promising target for vaccine development [11].

Nef is incorporated into viral particles and expressed in the early stage of infection both in the cytoplasm and on the cell membrane of virus-infected cells. Nef interacts with multiple host factors in order to optimise the cellular environment for virus replication [12]. Its critical role for viral pathogenicity is demonstrated by the fact that the infection with *nef*-defective HIV strains dramatically decreases the rate of disease progression in seropositive individuals [13]. Moreover, Nef is an important component for CTL-based HIV-1 vaccines. For this reason immune responses directed against this viral protein could help to control the initial steps of viral infection and to reduce viral loads and spreading [11].

In vitro proteolysis experiments have shown that Nef consists of an N-terminal membrane anchor region and a well folded C-terminal core domain [14]. The N-terminal membrane anchor domain structure has been solved in its myristoylated and non-myristoylated forms showing a flexible polypeptide chain with two helical structure elements [15].

When translated *in vitro*, the Nef gene yields two main polypeptides: a full-length N-terminal myristoylated form of 27 kDa (p27) and a truncated form of 25 kDa (p25) translated from a second start codon of the Nef gene and lacking the first 18 amino acids. Non-myristoylated p27 Nef mutant and p25 Nef were both found in the cytoplasm, while the wild-type, presumably myristoylated p27 Nef was mainly membrane associated [16]. Both p27 and p25 have been expressed in different biological systems. While the levels of p27 non-myristoylated expression in *E. coli *are reasonably high [17], protein yield in yeast and insect cells is very poor [18]. In particular, from the analysis of subcellular localization of the recombinant protein in yeast, it appears that the myristoylated form of Nef causes cell membranes perturbation [19]. Moreover, it has been shown that Nef expressed in transfected mammalian cell lines can be cytotoxic and cytostatic [20].

To explore the possibility of Nef expression in plants, we attempted a number of different strategies. We designed a panel of constructs to direct the expression of Nef polypeptides to either the cytosol or the secretory pathway. We tested these constructs initially by transient expression in tobacco protoplasts, to rapidly ascertain the most promising strategy for Nef production in stable transgenic plants. Plant protoplast transfection is an established *in vivo *approach that allows rapid assessment of heterologous protein expression in plant cells [21]. Moreover it allows for an accurate assessment of the intracellular fate of cytosolic or ER targeted recombinant proteins [22]. Here, we report the expression of the p25 and p27 HIV Nef polypeptides and of their non-myristoylated p27 variants.

## Results

### Nef is stable in the plant cytosol

**Figure 1:**
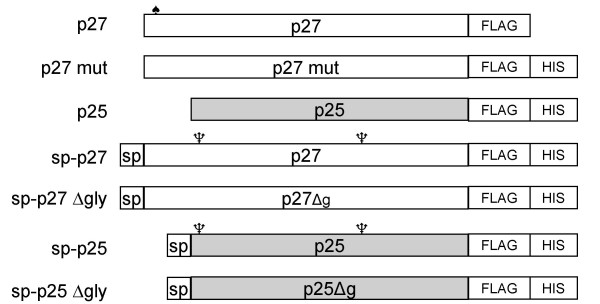
Diagram of the constructs used in this study. All Nef variants were under control of the CaMV 35S promoter. Sp: PR1 signal peptide; club: myristoylation site; tridents: predicted glycosylation sites; Δg: unglycosylated Nef forms. P27: *nef *full-length cDNA; p27 mut: mutated form of the p27 cDNA in which the myristoylation consensus sequence is abolished; p25: *nef *cDNA encoding truncated Nef.

**Figure 2:**
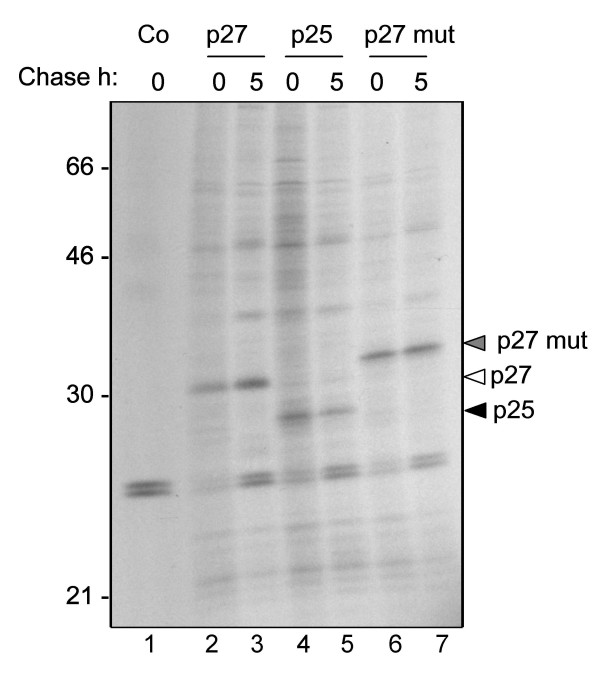
Nef is expressed and is stable in the cytosol of tobacco protoplasts. Tobacco mesophyll protoplasts were transfected with plasmids encoding the p27, p25 and p27 mut Nef variants, or with empty vector (Co). Transfected cells were labelled for 1 h with ^35^S methionine and cysteine and chased for 5 h with unlabelled amino acids. Cell homogenates were subjected to immunoprecipitation with anti-FLAG antiserum, followed by SDS-PAGE and fluorography. Numbers at left indicate molecular weight markers in kDa.

We set out to evaluate the ability of plant cells to cope with the synthesis of Nef. We generated a number of constructs for Nef expression in the plant cytosol or secretory pathway (Fig. 1). To facilitate immunodetection of the viral polypeptides, all constructs bore the FLAG and 6× histidine epitope tags at their C-termini, with the exception of wild-type p27 Nef that only carried the FLAG tag. We tested these constructs by transient expression in tobacco mesophyll protoplasts followed by pulse-chase analysis. We initially expressed the cytosolic forms of Nef: full length, both with (p27) or without (p27 mut) its N-terminal myristoylation signal, and truncated form (p25). Transfected protoplasts were metabolically labelled with ^35^S-methionine and ^35^S-cysteine and chased for 5 hours. Protoplast homogenates were then subjected to immunoprecipitation with anti FLAG antiserum. SDS-PAGE and fluorography revealed that all three constructs are successfully expressed, yielding polypeptides of the expected sizes (Fig. 2). Note that p27 is faster migrating than p27 mut as it does not contain the additional histidine tag (Fig. 2, compare lanes 4–5 with lanes 2–3). All proteins appeared to be stable over the course of the 5-hour chase.

**Figure 3:**
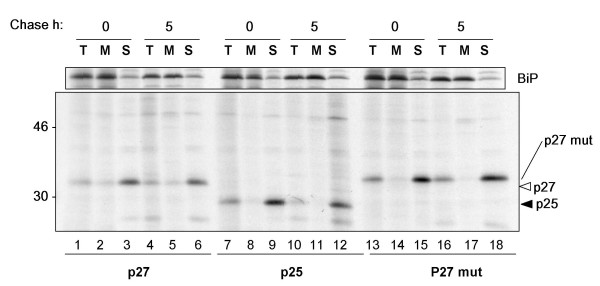
Cytosolic Nef variants are predominantly soluble. Protoplasts transfected with the indicated plasmids were pulse labelled for 1 h and chased for 5 h. Cells were then homogenised in 12% (w/w) sucrose and homogenates were fractionated by centrifugation at 100,000 g for 30 min on a 17% sucrose pad. Total homogenates (T), membrane pellets (M) and soluble supernatant (S) fractions were then immunoprecipitated with anti BiP (upper panel) or anti FLAG (lower panel) antisera. Numbers at left indicate molecular weight markers in kDa.

We wanted to investigate whether p27 was actually myrystoylated and able to interact with membranes. We therefore transfected protoplasts with plasmids encoding p25, p27 and p27 mut. After labelling, protoplasts were homogenised in 12% sucrose and total membranes pelleted. Most of the immunoselected Nef polypeptides were retrieved in the soluble fraction (Fig. 3, lower panel, S). As a control for microsomal membrane integrity, the endoplasmic reticulum-resident chaperone BiP (Fig. 3, upper panel) was mainly immunoselected from the membrane fractions (M). A slightly higher proportion of p27 was found in the membrane fraction than p27 mut (Fig. 3, compare lanes 2 and 5 with lanes 14 and 17). This indicates that a very small amount of p27 may indeed be membrane-associated, possibly via myristoylation. In any case, it appears that the vast majority of plant-made Nef is soluble and therefore unlikely to bear lipid modifications.

### Nef is unstable in the plant secretory pathway

**Figure 4:**
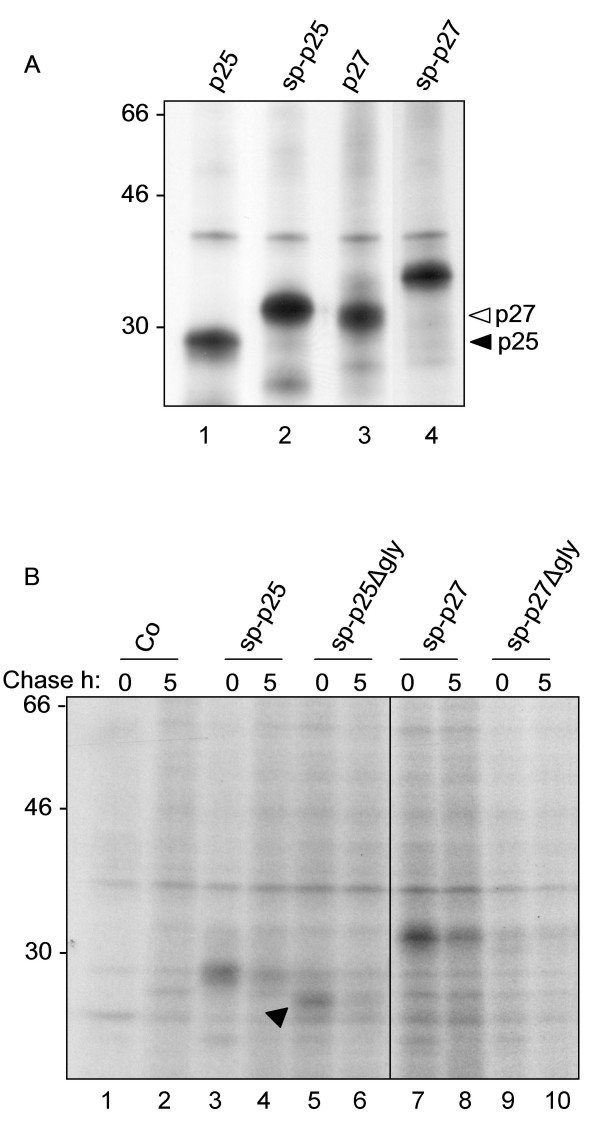
sp-Nef constructs are unstable in tobacco protoplasts. A: Protoplasts transfected with the indicated plasmids were pulse labelled for 2 h. Cell homogenates were subjected to immunoprecipitation with anti-FLAG antiserum, followed by SDS-PAGE and fluorography. Note the size difference between cytosolic p25 and p27 (as indicated by arrowheads) and their signal-peptide fusion counterparts. Numbers at left indicate molecular weight markers in kDa. B: Protoplasts transfected with the indicated plasmids were pulse labelled for 1 h and chased for 5 h. Cell homogenates were subjected to immunoprecipitation with anti-FLAG antiserum, followed by SDS-PAGE and fluorography. Arrowhead: aglycosylated Nef p25 (sp-p25Δg).

Having established that Nef is stable when expressed in the cytosol, we tested whether it was possible to express Nef in the secretory pathway. Therefore we fused the cDNAs for both p27 and p25 to the signal peptide (sp) of the PR1 protein [23] (constructs sp-p27 and sp-p25, Fig. 1). We have previously used this peptide successfully to drive the expression of immunoglobulin chains in tobacco cells [21]. We expressed the signal peptide fusions in tobacco protoplasts and subjected cells to 2 hours continuous labelling followed by homogenisation, immunoprecipitation with anti-FLAG antiserum, SDS-PAGE and fluorography. Figure 4A shows that both sp-p25 and sp-p27 are expressed and that their mobility is significantly reduced as compared to the cytosolic forms (Fig. 4A, note the size shift between lanes 1–2 and 3–4). Analysis of the Nef polypeptide sequence reveals the presence of two sequons that would be recognised as glycosylation sites upon translocation in the lumen of the endoplasmic reticulum. The presence of these glycans could explain the different electrophoretic mobility of the sp-Nef fusions. In order to prevent glycosylation – which of course does not occur during HIV infection – from interfering with the folding of the normally cytosolic, unglycosylated Nef protein, we inactivated the glycosylation sites by mutagenesis (constructs sp-p27Δgly and sp-p25 Δgly Fig. 1). We expressed these constructs in tobacco protoplasts and subjected cells to a 1-hour pulse labelling, followed by a 5-hour chase (Fig. 4B). Remarkably, both sp-p25 and sp-p27 resulted highly unstable, with their levels decreasing sharply during the 5-hour chase period (Fig. 4B, lanes 3–4 and 7–8). sp-p25Δgly showed increased mobility, as expected by removal of the glycosylation sites (Fig 4B, compare lanes 3–4 with lanes 5–6), but also disappeared during the chase. Expression of p27Δgly was barely detectable from the outset (Fig. 4B, lanes 9–10). None of the sp-Nef polypeptides appeared with time in the protoplast incubation medium (data not shown), indicating that sp-Nef fusions are capable of entering the secretory pathway (as indicated by glycosylation) but are not at all secreted. This phenotype – lack of secretion and fast intracellular degradation – indicates that the Nef polypeptides may be subject to strict quality control and subsequently disposed of by the secretory pathway [24-27]. Certainly the amount of 'secretory' Nef recovered after the chase did not compare favourably with the levels of cytosolically expressed protein. Targeting of Nef to the plant secretory pathway therefore proved to be a less successful strategy than cytosolic expression. For this reason we employed the cytosolic construct to generate stable transgenic tobacco plants.

### Stable Nef expression in transgenic tobacco and affinity purification

The sequences encoding p25 and p27 mut HIV-1 Nef variants were cloned into the binary plant expression vector pBI121 and the constructs p25 and p27 mut (Fig.1) used to generate transgenic tobacco plants. The putative transgenic tobacco plants were analysed by polymerase chain reaction (PCR) to verify the nuclear integration of *nef *gene (data not shown). Seventy p25 and 118 p27 mut lines were analysed, among these 67 and 115 respectively were positive for *nef *gene (data not shown).

**Figure 5:**
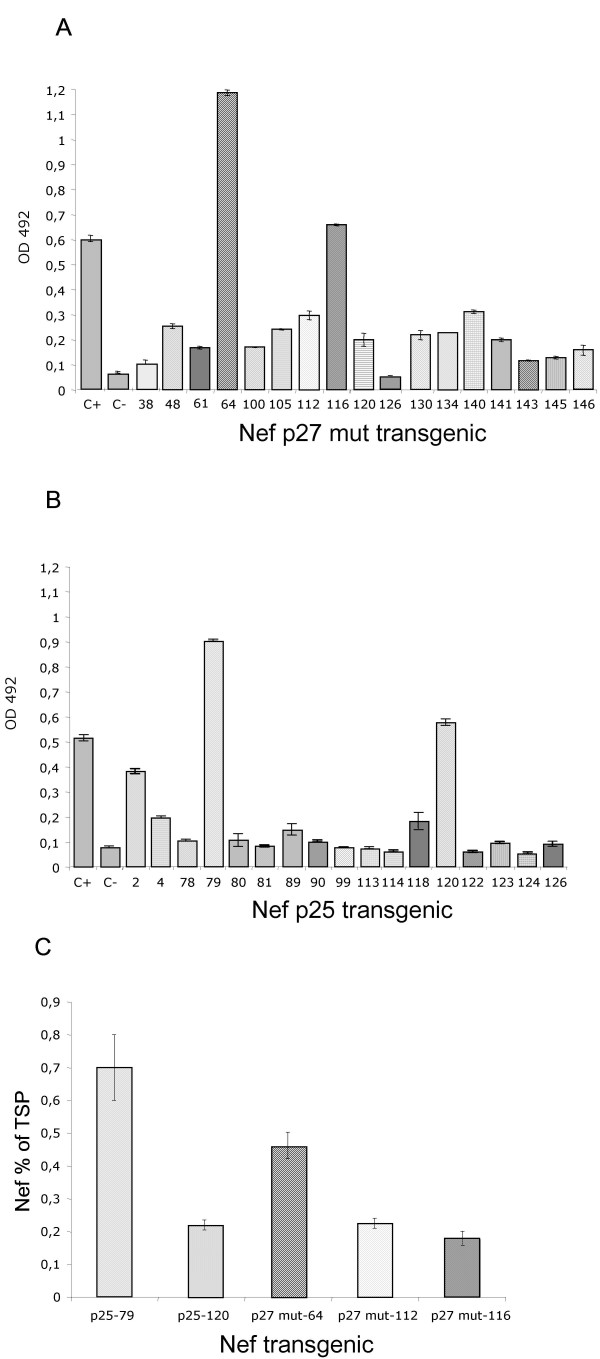
ELISA analysis of transgenic tobacco plants expressing cytosolic Nef. A:Representative ELISA assay of p27 mut Nef tobacco lines. C+: 100 ng of *E.coli *recombinant Nef; C-: control plant, untrasformed tobacco. The numbers on the x axis indicate individual transgenic tobacco lines. B:Representative ELISA assay of p25 Nef tobacco lines. C+: 100 ng of *E.coli *recombinant Nef; C-: control plant, untrasformed tobacco. The numbers on the x axis indicate individual transgenic tobacco lines. C: ELISA quantification of plant-expressed Nef variants. The Nef expression levels are expressed as percentage of total soluble protein (TSP) from transgenic plants. The values shown represent averages of two experiments. The numbers on the x axis indicate individual transgenic tobacco lines.

We analysed the levels of Nef protein expression by direct ELISA, using as primary antibody the antiserum to HIV-1 Nef (Fig. 5A–B). In these assays *E.coli *recombinant Nef was used as positive control. Fifteen p27 mut and 6 p25 lines were found to express Nef variants in detectable amounts. Among these, 3 lines expressing p27 mut (Fig. 5A, lines 64, 112, 116) and two lines expressing p25 (Fig. 5B, lines 79, 120) were further analysed to estimate Nef expression levels. We estimated the overall amounts of plant recombinant Nef – expressed as percentage of total soluble plant protein (TSP) – by using a standard curve generated with different concentrations of *E.coli*-produced recombinant Nef. As shown in Fig. 5C, the quantitative ELISA revealed variable expression levels of p25 and p27 mut Nef variants in TSP from individual transgenic lines ranging between 0.18% of TSP (Fig. 5C, p27 mut-116) and 0.7% (Fig. 5C, p25–79).

**Figure 6:**
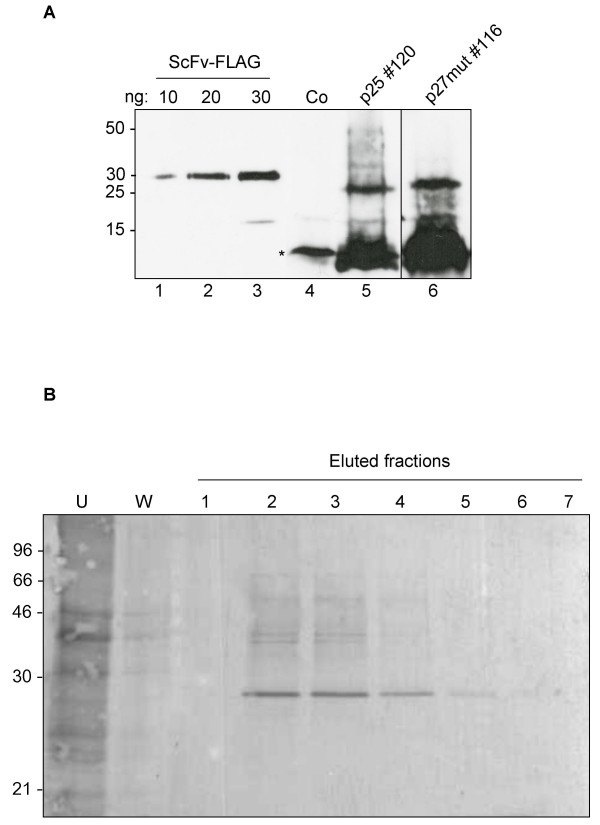
Analysis and affinity purification of plant-produced cytosolic Nef. A: Samples of leaf protein homogenates from a p25 (line 120) and a p27 mut (line 116) transgenic line were resolved by 15% SDS-PAGE and subjected to immunoblot with anti-FLAG polyclonal antiserum. A dilution series of a recombinant, FLAG-tagged ScFv [28] was loaded as a quantitative reference. The asterisk indicates a non-specific, anti FLAG – immunoreactive peptide. Numbers at left indicate molecular weight markers in kDa. Co: control mock-transformed plant. B: Total leaf protein homogenate from transgenic plant line 116 expressing p27 mut was subjected to affinity chromatography with a cobalt resin. His-tagged Nef was eluted with imidazole. U: fraction not bound to the resin; W: wash fraction. Numbers at left indicate molecular weight markers in kDa.

In order to assess the integrity of the plant-expressed Nef polypeptides, we collected tissue samples from plants expressing p25 or p27 mut and subjected them to SDS-PAGE alongside increasing concentrations of a FLAG-tagged anti-lysozyme ScFv [28] as a quantitative control (Fig. 6A). Immunoblot with anti FLAG antiserum revealed an immunoreactive polypeptide of the size predicted for both p25 and p27 mut and showed clear accumulation of the proteins. In addition, a large amount of faster migrating immunoreactive peptides were also detected. As these are not present in samples from control plants containing empty vector, they are likely to be degradation products of the Nef polypeptide.

We took advantage of the 6× histidine tag appended to p27 mut to perform a small-scale purification of Nef by loading homogenates from transgenic leaf sections on a cobalt affinity purification column. Immunoblot with anti FLAG antiserum (Fig. 6B) shows that the eluate from this one-step procedure yields an anti FLAG-immunoreactive polypeptide of the size expected for p27 mut. No degradation products are detected, indicating that the purified protein is stable.

## Discussion and conclusion

We have assessed a number of strategies for the expression of HIV Nef *in planta*. We initially used tobacco mesophyll protoplast transfection to test constructs encoding full-length Nef, with (p27) or without its myristoylation signal (p27 mut), or truncated Nef (p25). The use of protoplast transfection, coupled with metabolic labelling and pulse-chase experiments, allowed us to very rapidly determine what intracellular location was most suitable for expression of Nef. Our results indicate that cytosolic Nef polypeptides are correctly synthesised and relatively stable over a 5-hour time course. The same was not true for Nef polypeptides targeted to the secretory pathway by virtue of a signal peptide. Although the proteins translocated correctly into the lumen of the endoplasmic reticulum, as demonstrated by efficient N-glycosylation, they were not stable. Moreover, there was no evidence for secretion of any of the proteins. Even the removal of the glycosylation sites did not significantly improve stability, or promote secretion. Although redirection of cytosolic proteins into the lumen of the secretory pathway has often achieved high yield of recombinant protein [29-32] it is possible that Nef, which is normally located in the cytosol or in association with the cytosolic face of the plasma membrane [16], does not fold correctly within the milieu of the endoplasmic reticulum. The fact that no polypeptides are secreted and that degradation seems to occur very rapidly indicate that Nef may be disposed of by ER quality control mechanisms [26,33].

Our data indicate that cytosolic expression is a more promising strategy. We therefore generated transgenic plants expressing full length or truncated Nef. Expression levels were variable, but in some lines they averaged 0.7% of total soluble proteins. This is in line with a number of other heterologous proteins expressed in transgenic tobacco [34]. Moreover, it is possible that the ELISA values represent an underestimate. We have indeed found that plant protein homogenates partially interfere with the detection of recombinant Nef in ELISA assay: by mixing recombinant *E.coli *HIV-1 Nef with control plant protein extracts, we often observed a significant reduction in the ELISA reading as compared with the Nef protein in buffer alone (data not shown).

Interestingly, cytosolically expressed Nef is almost completely non-myristoylated, as indicated by poor membrane partitioning of Nef p27. This is somewhat surprising, as the N-terminal myristoylation machinery is conserved in plants [35,36]. In any case, the lack of myristoylation is actually beneficial to the immunogenic properties of Nef for the application in a multi-component vaccine [37]. Indeed, deletion or mutagenesis of the N-terminal myristoylation site has been shown to abrogate the capacity of Nef to down-regulate both MHC class I and CD4 cell-surface molecules [38], which normally prevents CTL-mediated lysis of HIV-1-primary infected cells [39]. Therefore the lack of myristoylation is likely to elicit enhanced cellular immune responses.

Plant-expressed Nef could be purified easily from transgenic leaves using a cobalt affinity column. This indicates that the hexahistidine tag is correctly exposed in the plant-made polypeptides. This ease of purification, together with our data showing that cytosolic Nef expression levels *in planta *are satisfactory, constitutes a useful starting point for further optimisation and scale-up of expression. This will allow us to analyse the biological activity and *in vitro/in vivo *immunological properties of plant-produced Nef proteins.

## Methods

### Recombinant DNA

The pSCNef51 (ARP#2015 NIBSC-CFAR MRC) plasmid was used as the source of Nef cDNA (HIV-1 BH10 strain). In all constructs (Figure 1) Nef encoding sequences were under the control of the constitutive CaMV 35S promoter and the nopaline synthase (NOS) terminator sequence. To enhance translational efficiency, the Tobacco Mosaic Virus (TMV) 5' leader sequence Ω [40] was fused in frame with the Nef sequences. Moreover to facilitate the purification and detection of the recombinant proteins, all Nef coding sequences harbour at their 3'end the sequences encoding the FLAG and polyhistidine-tag peptides, with the exception of wild-type p27 Nef that only carried the FLAG tag. All inserts were generated by overlap extension PCR [41]. Briefly, a set of primers was used to generate by PCR, using Pfu polymerase (Stratagene, La Jolla, CA), two DNA fragments having overlapping ends. PCR-generated DNA fragments were first purified from agarose gel using GFX PCR DNA and Gel Band Purification Kit (Amersham Bioscience) and then employed in a subsequent overlap extension reaction to obtain the resulting fusion products. To amplify the fusion products cloned in the constructs p25 and p27 mut (Figure 1) the following forward primers were used BNFor (5' CGGGATCCATGAGACGAGCTGAGCCAGCAGCAGATG 3') and BNMFor (5' CGGGATCCATGGCCGGCAAGTGGTCAAAAAGTAGTGTG 3'). The HSRev (5' TCCCCCGGGCTAATGGTGATGGTGATGGTGCTTGTCGTC 3') was used as the reverse primer. P27 mut construct contains a mutated form of the p27 Nef, coding for a full-length protein in which the myristoylation consensus sequence is abolished by the substitution of the glycine residue in second position into alanine. P25 and p27 mut HIV Nef variants were cloned in the binary vector pBI121. To allow transient expression by protoplast transfection, the DNA cassettes starting from the CaMV 35S promoter and ending with the NOS terminator sequence (Figure 1) were cloned into the vector pGEM^R^-4Z vector for transient expression.

For generation of the p27 construct, the Nef coding sequence was amplified from plasmid pSCNef51 using the P27 Xba1 Forward (5' CAGAGTCGTCTAGAGGTGGCAAGTGGTCAAAAAGT 3') and Nef Flag Reverse (5' 3') primers. The resulting PCR product was cloned into the pDHA vector [42] for transient expression.

The PR1 signal peptide was amplified from plasmid pDE300d [23] using the sPR1BamH1Forward (5' CAGAGTCGGGATCCATGGGATTTTTTCTCTTTTC 3') and sPR1Xba1Reverse (5' CAGAGTCGTCTAGACGCATGAGAAGAGTGAG 3') primers. To amplify the sp-Nef variants for cloning into pDHA, the following forward primers were used: p25Xba1For (5' CAGAGTCGTCTAGAAGACGAGCTGAGCCAG 3') and P27Xba1For (5' CAGAGTCGTCTAGAGGTGGCAAGTGGTCAAAAAGT 3'). The NefPst1Rev (5' CAGAGTCGCTGCAGCTAATGGTGATGGTGATG 3') was used as the reverse primer.

### Plant transformation

The constructs p25 and p27 mut were electroporated into *A.tumefaciens *strain LBA4404. Leaf discs of *Nicotiana tabacum *cv Petit Havana SR1, were transformed according to the protocol described elsewhere [43].

### Protoplast transfection

Protoplasts prepared from axenic leaves of tobacco were subjected to polyethylene glycol-mediated transfections exactly as described by [33]. 40 μg of each plasmid was used to transform 10^6 ^protoplasts in 1 ml. After transfection, cells were incubated for 16 h at 25°C before metabolic labelling.

### In vivo labelling of protoplasts and analysis of expressed polypeptides

Pulse-chase experiments, immunoprecipitation, SDS-PAGE and fluorography were performed as described previously [21]. Immunoprecipitation was performed with anti FLAG antiserum (Sigma, 1:1000) or anti Nef (EVA#3067.4, NIBSC-CFAR MRC) at a 1:1000 dilution.

### ELISA detection and quantification of plant-expressed Nef variants

Expression of p25 and p27 mut Nef variants, in tobacco plants, were determined by direct ELISA. TSP were obtained as described: for each sample, 100 mg of leaf tissue was ground thoroughly in liquid nitrogen and the powder was added with 500 μl of PBS-buffer (PBS) containing protease inhibitors (Complete EDTA Free, ROCHE). Extracts were clarified by centrifugation at 20000 × g for 30 min at 4°C. ELISA assays were performed coating 96-well microplates with 100 μl of total protein extract for each sample, for 2 h at 37°C or O/N at 4°C. *E.coli *recombinant Nef (EVA#650, NIBSC-CFAR MRC) was used as positive control. The plates were blocked with 5% milk/PBS at 37°C for 2 h. After washing, plant recombinant Nef was detected using the HIV-1 Nef rabbit Antiserum (Cat#2949 NIH AIDS Research and Reference Reagent Program) [44] diluted 1:100 in PBS 2% milk, at 37°C for 2 h or O/N at +4°C, followed by incubation with Biotin-Labeled Affinity Purified anti-rabbit IgG (Cat#16-15-06 KPL) diluted 1:2500 and strepavidin-horseradish peroxidase conjugate (RPN 1231 Amersham) diluted 1:2000, at 37°C for 2 h. The substrate was OPD (AGDIA kit). The reaction was stopped after 1 h, by addition of 1/2 volume of 3 M H_2_SO_4 _and the colorimetric reaction was measured at 492 nm. Plant recombinant Nef levels, expressed as% TSP, were estimated using, as standard curve, different concentrations (ranging between 100 and 12.5 ng) of E.coli recombinant Nef (EVA#650, NIBSC-CFAR MRC). The amount of total soluble proteins (TSP) was estimated by Bradford assay. The OD value from each sample was subtracted from control plant OD value.

### Immunoblot analysis of expressed polypeptides

Leaf samples (10 mg) were ground in 50 μl of 1× SDS-PAGE sample buffer and centrifuged at 12,000 × g for 10 min. The supernatants were boiled and loaded on 15% SDS-PAGE (w/v) followed by immunoblotting with FLAG antiserum (Sigma, UK). Signal was detected by ECL Plus (Amersham).

### Cobalt affinity purification of 6× histidine-tagged Nef

Leaves of a transgenic tobacco plant expressing p27 mut were harvested and ground in liquid nitrogen. The sample was thawed in the presence of lysis buffer (20 mM Tris-HCl pH 8.0, 100 mM NaCl) + protease inhibitors (Complete EDTA-free, Roche). The sample was centrifuged at 12,000 × g for 30 minutes at 4°C to pellet the debris. Purification was carried out in a batch method as detailed in the manufacturer's instructions. Briefly, the clarified sample was added to 2 mls of Talon™ cobalt resin (Clontech) sand mixed at room temperature for 30 minutes. The resin was washed twice with 10 column volumes of lysis buffer before being added to a plastic column. The resin was washed with 2 column volumes of wash buffer (lysis buffer + 10 mM imidazole). Proteins were eluted in 1 ml fractions by the addition of elution buffer (lysis buffer + 50 mM imidazole). Fractions were analysed by SDS-PAGE followed by immunoblotting with Flag antiserum (Sigma, UK).

## Authors' contributions

**CM **generated all expression constructs and contributed to the development of the experimental strategy and to the writing of the manuscript; **JN **generated the wild-type p27 clone and performed all transient expression experiments; **GB **performed ELISA detection and quantification of plant-expressed Nef; **CL **contributed to generate expression constructs; **RL **performed PCR analysis of transgenic tobacco plants; **SB **contributed to the development of experimental strategy; **EB **is ENEA group coordinator and contributed to the development of experimental strategy; **LF **conceived the transient expression part of the project and wrote the manuscript.

All authors read and approved the final manuscript.
